# Misdiagnosis of ANCA‐Associated Vasculitis in Patients With Cocaine/Levamisole–Associated Autoimmune Syndrome and Cocaine‐Induced Midline Destructive Lesions: A Case Series

**DOI:** 10.1002/iid3.70215

**Published:** 2025-06-17

**Authors:** Kehinde Sunmboye, Ameen Jubber, Maumer Durrani, Jeremy Royle, Shireen Shaffu

**Affiliations:** ^1^ University of Leicester Leicester UK; ^2^ University Hospitals of Leicester Leicester UK

**Keywords:** ANCA‐associated vasculitis, Cocaine, Cocaine/Levamisole‐associated autoimmune Syndrome, granulomatosis with polyangiitis, misdiagnosis, nasal septum perforation

## Abstract

**Background:**

Cocaine/Levamisole‐Associated Autoimmune Syndrome (CLAAS) encompasses a spectrum of autoimmune and vasculitic phenomena, which includes Cocaine‐Induced Midline Destructive Lesions (CIMDL), which can mimic ANCA‐associated vasculitis (AAV) due to overlapping clinical features and the potential for ANCA positivity. These similarities can lead to misdiagnosis and inappropriate immunosuppressive therapy.

**Methods:**

This study highlights a case series of seven patients (from 2015 to 2024) with CLAAS with its subset of CIMDL, initially misdiagnosed as active AAV, in patients who were referred to various clinicians in the Rheumatology unit of a Tertiary Hospital in the United Kingdom.

**Results:**

All patients presented with nasal symptoms, and they all exhibited additional systemic manifestations consistent with CLAAS. Five were ANCA‐positive at initial evaluation, leading to the initiation of immunosuppressive therapy; however, symptoms persisted. The diagnoses were then revised to CIMDL in all cases within the broader context of CLAAS following the identification of cocaine use after further patient inquiry and urine toxicology for drug of abuse (DOA) screening found cocaine metabolites.

**Conclusion:**

A comprehensive drug history and urine toxicology screening are crucial in patients with suspected AAV, as ANCA positivity can occur in CLAAS as well as its subset of CIMDL, complicating the diagnosis. Differentiating between AAV and CIMDL related to CLAAS is essential to avoid unnecessary immunosuppression.

## 1 Introduction

The emergence of cocaine/levamisole‐associated autoimmune syndrome (CLAAS), alongside its subset of cocaine‐induced midline destructive lesions (CIMDL), has significantly complicated the diagnostic landscape, particularly in the evaluation of patients presenting with symptoms suggestive of vasculitis. These syndromes introduce new challenges due to the clinical overlap between drug‐induced conditions and genuine autoimmune disorders, such as antineutrophil cytoplasmic antibody (ANCA)‐associated vasculitis (AAV), most notably granulomatosis with polyangiitis (GPA). The shared clinical manifestations between CIMDL and AAV—such as nasal crusting, epistaxis, and septal perforation—make it difficult to distinguish between these two conditions based solely on clinical presentation [1, 2]; (Berman et al., 2016). CLAAS, which is a systemic manifestation of cocaine induced disease can also cause constitutional symptoms that can occur in AAV. CIMDL, the localized manifestation of CLAAS can create diagnostic dilemmas as it can cause epistaxis, nasal crusting and facial pain [1]. Patients with CLASS as well as CIMDL can also tests positive for ANCA, a serological marker traditionally associated with active AAV, particularly with the cytoplasmic ANCA (c‐ANCA) directed against proteinase‐3 (PR3), which can blur the diagnostic boundaries between drug‐induced and autoimmune processes [2, 3]. This overlap in clinical and serological findings often leads to misdiagnoses, which may result in the inappropriate administration of immunosuppressive therapies. These treatments, while essential for managing AAV, expose patients to unnecessary risks, including increased susceptibility to infections and other complications, when misapplied in cases of substance‐induced disease (Berman et al., 2016).

Levamisole, a common adulterant found in street cocaine, has been increasingly implicated in the pathogenesis of autoimmune‐like phenomena seen in both CLAAS and CIMDL, including ANCA positivity and vasculitis‐like syndromes. Levamisole can induce a variety of vasculitis‐like syndromes that closely mimic AAV, complicating the diagnostic process further (Graf et al., 2011; Cascio & Jen, 2018; Jin et al., 2018). The pathophysiological mechanisms underlying CLAAS as well as CIMDL, particularly the contributions of both cocaine and its adulterants like levamisole in inducing ANCA positivity, are areas of ongoing research that warrant careful clinical scrutiny [1]; (Graf et al., 2011; Cascio & Jen, 2018).

Studies have demonstrated that both cocaine and levamisole can activate neutrophils, leading to the formation of neutrophil extracellular traps (NETs), a process implicated in the promotion of inflammation and the development of autoimmunity [1]; (Cascio & Jen, 2018). The presence of levamisole in cocaine has been particularly associated with a distinctive clinical syndrome characterized by purpura, skin necrosis, and a high prevalence of autoantibodies, including ANCAs (Jin et al., 2018). This association between levamisole and autoimmune‐like phenomena adds to the diagnostic complexity, as patients often present with clinical features characteristic of both CIMDL and AAV, necessitating a nuanced and thorough evaluation [1, 2]; (Cascio & Jen, 2018).

In the context of therapeutic interventions, it is critical to recognize that patients with CIMDL frequently do not respond to conventional immunosuppressive therapies that are typically effective for AAV. This resistance to standard treatment further complicates patient management, as clinicians may continue to escalate immunosuppressive regimens in the mistaken belief that they are dealing with a refractory case of AAV [2]; (Berman et al., 2016). However, cessation of cocaine use has been shown to significantly improve clinical outcomes in patients with CLAAS as well as CIMDL, limiting disease progression and enhancing surgical recovery when necessary [4]. Therefore, a critical component of improving diagnostic accuracy and patient management involves taking a thorough drug use history, including detailed inquiries about cocaine (often with levamisole exposure), and incorporating routine urine toxicology screening into the diagnostic workup within the clinic setting.

This case series therefore aims to emphasize the importance of urine toxicology in the diagnostic process for all patients with AAV and optimize patient care by emphasizing the importance of distinguishing between drug‐induced vasculitis and true autoimmune vasculitis. We have also set out to see if there is any pattern of clinical or biochemical features unique to this cohort of patients. The discussion will also focus on key clinical and histopathological differences between CLAAS as well as CIMDL and AAV, as well as the patterns of ANCA positivity that may aid clinicians in making an accurate diagnosis. Additionally, this study underscores the need for a multidisciplinary approach, involving rheumatologists, otolaryngologists, and addiction specialists, to ensure that patients with suspected drug induced vasculitidies receive appropriate care and avoid the risks associated with misdiagnosis [5].

## 2 Case Series

### 2.1 Case 1: Patient A

#### 2.1.1 Case Summary

A 26‐year‐old woman presented with pulmonary hemorrhage, arthralgia affecting the small joints of her hands, and a history of constitutional symptoms. She also reported numbness and neuropathic pain in her limbs.

#### 2.1.2 Diagnostic Workup

**Figure 1:**
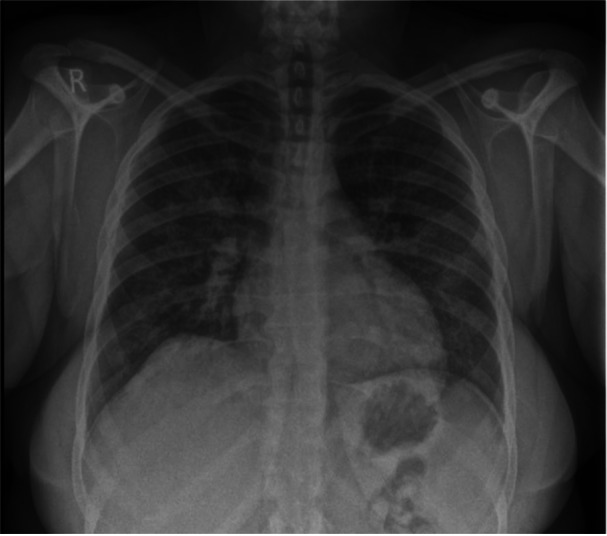
Chest x‐ray for patient A, showing Bilateral lung field haziness.

**Figure 2:**
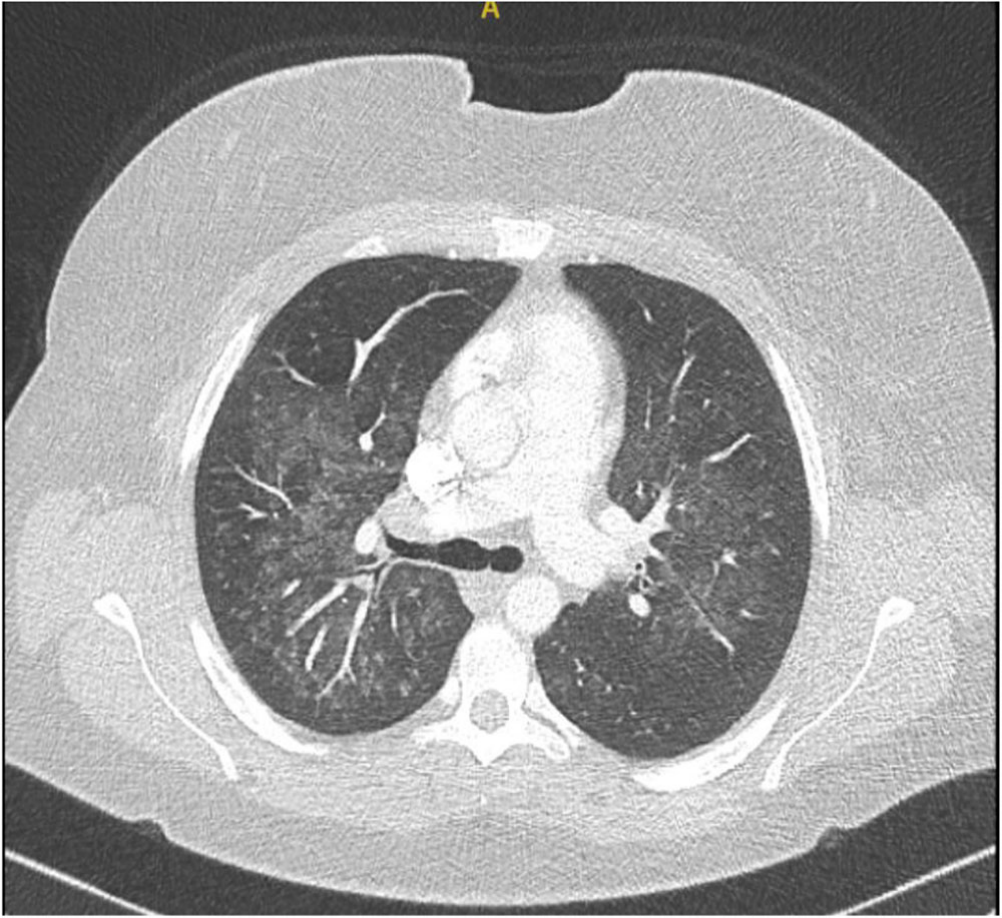
Chest CT for patient A showing ground glass shadowing at the level of the tracheal bifurcation.

The admitting respiratory team did not initially document a history of recreational drug use. Blood tests revealed elevated inflammatory markers without eosinophilia. A chest x‐ray showed bilateral haziness across the lung fields (Figure 1), and chest CT imaging confirmed the presence and extent of pulmonary involvement (Figure 2). The combination of radiographic changes and raised C‐reactive protein levels (see Table 1) prompted a provisional diagnosis of active ANCA‐associated vasculitis (AAV), although ANCA testing returned negative.

**Table 1:** Table showing patient demographics, renal function, blood eosinophilia, ANCA positivity and subtype and urine metabolite products.

Patient age (in years)	Gender	eGFR (ml/min/1.73 m^2^)	Eosinophila at presentation (Highest Value of Eosinophilia) Normal: less than 0.4 × 109/L)	C‐reactive protein level (mg/L) (Normal < 5)	ANCA result	Peak PR3‐ANCA levels (if applicable) Normal value for PR3‐ANCA ( < 3.5 IU/ml)	Abnormal Chest imaging (Chest x‐ray or CT chest)	Urine toxicology results	Current clinical outcome
26	Female	> 90	No	13	Negative	Not applicable	Yes	Benzoylecgonine, Cannabinoids	Discharge from Hospital care
31	Female	> 90	Yes (0.58)	Normal	Positive (C‐ANCA)	PR3‐ANCA (12 IU/ml)	No	Benzoylecgonine	Discharge from Hospital care
33	Female	> 90	Yes (0.52)	19	Positive (C‐ANCA)	PR3‐ANCA (119 IU/ml)	No	Benzoylecgonine and norcocaine	Under clinical surveillance not on treatment
34	Male	> 90	Yes (0.90)	Normal	Negative	Not applicable	No	benzoylecgonine, morphine, codeine, norcodeine and hydrocodone	Discharge from Hospital care
44	Female	> 90	Yes (1.09)	11	Positive (C‐ANCA)	PR3‐ANCA (40 IU/ml)	No	Pregabalin, benzoylecgonine and norcocaine, Tramadol and metabolite(o‐desmethyltramadol)	Discharge from Hospital care
47	Male	86	Yes (0.61)	16	Positive (C‐ANCA)	PR3‐ANCA (23 IU/ml)	No	Benzoylecgonine) and cannabinoid metabolite (THC‐COOH)	Discharge from Hospital care
58	Male	> 90	Yes (0.99)	31	Positive (C‐ANCA)	PR3‐ANCA (38 IU/ml)	Yes	Cocaine, Benzoylecgonine,	Under clinical surveillance on conventional synthetic disease modifying herapy

A more detailed social history later revealed regular cocaine use. Urine toxicology confirmed the presence of benzoylecgonine (a cocaine metabolite) and cannabinoids, establishing recent drug exposure.

#### 2.1.3 Treatment and Outcome

Clinicians initially administered high‐dose intravenous methylprednisolone for suspected AAV. However, following confirmation of cocaine exposure, they revised the diagnosis to pulmonary hemorrhage secondary to CLAAS. The patient's symptoms resolved without the need for further immunosuppressive therapy. She did not require ongoing specialist follow‐up. The team provided education about the health risks of continued drug use and referred her to addiction support services.

### 2.2 Case 2: Patient B

#### 2.2.1 Case Summary

A 31‐year‐old woman presented with several months of recurrent nasal crusting and episodes of epistaxis. She also reported bilateral sensorineural hearing loss and arthralgia affecting the small joints of her hands.

#### 2.2.2 Diagnostic Workup

Her laboratory tests showed normal inflammatory markers but revealed eosinophilia on her full blood count. Chest imaging was unremarkable. A nasal biopsy demonstrated moderately severe inflammation, raising suspicion for ANCA‐associated vasculitis (AAV). Serological testing confirmed elevated PR3‐ANCA levels at 12 IU/mL (normal < 3.5 IU/mL) (see Table 1). At the time of initial evaluation, the team did not obtain a history of recreational drug use.

#### 2.2.3 Treatment and Outcome

She began immunosuppressive treatment with methotrexate, which was later switched to azathioprine due to persistent symptoms. As her condition remained refractory, clinicians escalated treatment to rituximab. Despite this, she continued to experience nasal crusting, recurrent epistaxis, and worsening joint pain.

A regional specialist center subsequently reviewed her case. Urine toxicology testing detected benzoylecgonine, confirming recent cocaine use. This led to a reassessment of her diagnosis, which was revised to CIMDL. Her clinical team discontinued rituximab, and she has since remained off immunosuppressive therapy. She received counseling about the health risks of ongoing drug use and was referred to addiction support services.

### 2.3 Case 3: Patient C

#### 2.3.1 Case Summary

A 33‐year‐old woman presented with nasal crusting, a history of nasal septal perforation, and polyarthralgia. She had previously received ENT care for her nasal symptoms but was referred to the rheumatology team due to persistent and refractory issues.

#### 2.3.2 Diagnostic Workup

Her blood tests showed elevated inflammatory markers and eosinophilia on full blood count. Serological testing revealed a markedly elevated PR3‐ANCA level of 119 IU/mL (normal < 3.5 IU/mL). The rheumatology team performed a routine urine toxicology screen, which detected cocaine and its metabolites—benzoylecgonine and norcocaine. Following these results, the patient disclosed a history of recreational drug use.

#### 2.3.3 Treatment and Outcome

Although the referral was based on a working diagnosis of active ANCA‐associated vasculitis, the combination of persistent nasal disease and positive toxicology findings prompted a revision of the diagnosis to CIMDL. She continues treatment with methotrexate for ongoing inflammatory joint symptoms and nasal involvement. However, her management has not escalated to rituximab. The team provided counseling on the health risks associated with continued drug use and referred her to addiction support services.

### 2.4 Case 4: Patient D

#### 2.4.1 Case Summary

A 34‐year‐old man presented with several years of nasal congestion that improved with both inhaled and oral corticosteroids. He also experienced purulent nasal discharge, a sensation of nasal fullness, and worsening symptoms when steroids were withdrawn. He had a history of recurrent visits to ENT services for nasal obstruction and intermittent sinus blockage.

#### 2.4.2 Diagnostic Workup

Laboratory tests showed normal inflammatory markers and eosinophilia on full blood count. A nasal biopsy demonstrated moderately severe inflammation but no histological evidence of vasculitis. His initial ANCA result was positive but became negative on repeated testing. Urine toxicology identified multiple drug metabolites, including benzoylecgonine (cocaine), morphine, codeine, norcodeine, and hydrocodone, raising strong suspicion of drug‐induced nasal injury consistent with CIMDL.

#### 2.4.3 Treatment and Outcome

Clinicians had initially treated him with corticosteroids based on a presumed diagnosis of limited ANCA‐associated vasculitis. However, the confirmation of recent cocaine use prompted a diagnostic revision to CIMDL. His team discontinued steroids and transitioned to conservative management. He was counseled about the health risks associated with ongoing drug use and referred to addiction support services.

### 2.5 Case 5: Patient E

#### 2.5.1 Case Summary

A 44‐year‐old woman presented with a nasal septal perforation. She had a known diagnosis of multiple sclerosis and was receiving natalizumab infusions as part of her treatment. She also reported ongoing nasal symptoms, including nasal crusting and dryness.

#### 2.5.2 Diagnostic Workup

Blood tests revealed slightly elevated inflammatory markers and eosinophilia on full blood count. Further serological testing showed a positive PR3‐ANCA result at 40 IU/mL (normal < 3.5 IU/mL). Urine toxicology confirmed the presence of multiple drug metabolites, including benzoylecgonine and norcocaine (cocaine metabolites), as well as tramadol, o‐desmethyltramadol, and pregabalin.

#### 2.5.3 Treatment and Outcome

Although she was initially evaluated for possible ANCA‐associated vasculitis, the detection of cocaine use on toxicology testing led to a revised diagnosis of CIMDL. Her clinical team did not initiate immunosuppressive therapy. She received counseling on the risks of continued drug use and was referred to addiction support services.

### 2.6 Case 6: Patient F

#### 2.6.1 Case Summary

A 47‐year‐old man presented with persistent nasal crusting and epistaxis, along with polyarthralgia affecting multiple joints.

#### 2.6.2 Diagnostic Workup

Blood tests showed elevated inflammatory markers and eosinophilia on full blood count. Serology confirmed a persistently positive PR3‐ANCA at 23 IU/mL (normal < 3.5 IU/mL). At the time of initial assessment, clinicians did not obtain a social drug history.

#### 2.6.3 Treatment and Outcome

He received multiple immunosuppressive therapies, including methotrexate, azathioprine, and mycophenolate mofetil. Due to lack of response, his treatment was escalated to rituximab. Despite ongoing rituximab and corticosteroid therapy, his nasal and systemic symptoms worsened.

A urine toxicology screen was eventually performed and detected benzoylecgonine (cocaine metabolite) and THC‐COOH (cannabinoid metabolite). Following this result, he disclosed ongoing cocaine use. Clinicians revised his diagnosis to CLAAS with associated CIMDL. Rituximab and corticosteroids were discontinued. He remains on mycophenolate mofetil for management of CLAAS. He was counseled on the risks of continued drug use and referred to addiction support services.

### 2.7 Case 7: Patient G

#### 2.7.1 Case Summary

**Figure 3:**
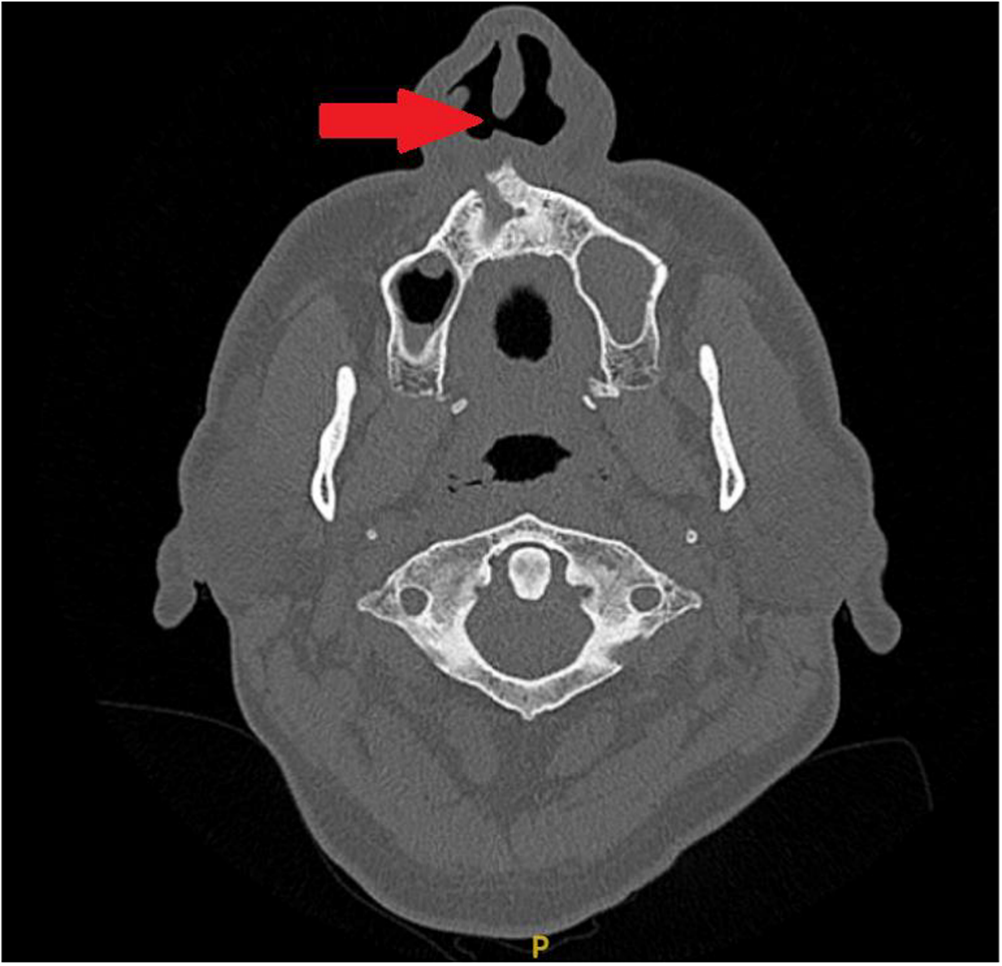
CT sinus (axial view), with the red arrow showing nasal perforation.

A 58‐year‐old man presented to ENT services with several years of recurrent sinusitis, nasal discharge, crusting, and epistaxis. He also reported polyarthralgia and had a documented history of ocular inflammation, specifically uveitis. CT imaging of the sinuses revealed a nasal septal perforation (Figure 3).

#### 2.7.2 Diagnostic Workup

Laboratory testing showed elevated inflammatory markers and eosinophilia on full blood count. Serological analysis revealed a positive PR3‐ANCA result at 38 IU/mL (normal < 3.5 IU/mL). At the time of initial evaluation, the clinical team did not obtain a social drug history.

#### 2.7.3 Treatment and Outcome

Based on his ocular involvement and positive ANCA serology, clinicians initiated treatment with rituximab for suspected ANCA‐associated vasculitis (AAV). Despite therapy, he remained symptomatic, and his PR3‐ANCA titers continued to rise.

A urine toxicology screen later detected both cocaine and its metabolite benzoylecgonine. In light of this finding, clinicians attributed his persistent ANCA positivity to ongoing cocaine exposure. They discontinued rituximab and administered intravenous methylprednisolone to manage acute symptoms. No additional immunosuppressive agents were started. He received counseling regarding the impact of cocaine use on his health and was referred to addiction support services.

## 3 Discussion

The cases presented highlight the complex diagnostic challenges involved in distinguishing CLAAS and CIMDL from primary ANCA‐associated vasculitis (AAV). This challenge is especially evident in patients who test positive for ANCA, a marker long considered central to diagnosing AAV. However, this assumption can mislead clinicians when assessing patients with recent or ongoing cocaine exposure [1, 2]. This is made even more imperative with the recent increase in recreational drug use in many countries in the world [6].

Overlapping features, such as nasal crusting, epistaxis, septal perforation, and constitutional symptoms, commonly appear in both CIMDL/CLAAS and AAV [7, 8]. Clinicians often attribute these signs to idiopathic vasculitis without exploring substance‐related causes. Recent studies [2, 7] have pointed out that the serologic distinctions between CIMDL and AAV, especially granulomatosis with polyangiitis (GPA) can be challenging. Gill et al. [2] reported that 87.5% of CIMDL patients are ANCA positive, with 56% showing PR3‐ANCA positivity and the rest exhibiting MPO‐ANCA positivity.

In several of the cases in this series, patients tested positive for PR3‐ANCA with cytoplasmic (C‐ANCA) patterns. One patient had a PR3‐ANCA titer of 119 IU/mL. While PR3‐ANCA is typically associated with granulomatosis with polyangiitis, its presence is increasingly observed in drug‐induced syndromes [9, 10]. This is especially true when atypical ANCA is picked up due to elastase antibodies being involved, a known feature of CIMDL and CLAAS (Cohen Tervaert & Damoiseaux, 2012 [11]). The presence of anti‐human neutrophil elastase (HNE) antibodies, though not routinely tested in all centers, can be invaluable in differentiating CIMDL from GPA in this scenario.

While the absence of elastase ANCA testing across this case series study represents a limitation, as pointed out human neutrophil elastase antibodies can help distinguish CIMDL and would have strengthened diagnostic accuracy in making a distinction between CIMDL/CLAAS and true AAV [1, 3]. Although P‐ANCA patterns with MPO‐ANCA or elastase specificity are more typical in CIMDL, five of the seven patients in this series showed C‐ANCA with PR3‐ANCA, further confounding the clinical picture. Some may have had primary AAV with incidental cocaine use, but others more likely had drug‐induced vasculitis that mimicked AAV serologically as they were consequently discharged from hospital care without clinical sequelae.

These serological overlap highlight the need to interpret ANCA results within the right clinical context. PR3‐ANCA alone should not confirm AAV in the absence of thorough drug history and toxicology testing (Cohen Tervaert & Damoiseaux, 2012 [9]). In all seven cases presented, the clinicians revised their diagnoses only after identifying cocaine metabolites such as benzoylecgonine, or other substances including cannabinoids, opioids, and tramadol, via urine toxicology when the clinical course of the patient and poor response to immunosuppressive therapy necessitated a review.

Omitting routine urine toxicology screening delayed accurate diagnosis and led to inappropriate immunosuppression in several of these patients. High‐dose corticosteroids, methotrexate, azathioprine, mycophenolate mofetil, and rituximab were used before drug exposure was confirmed. These treatments carry serious risks when misapplied when ignoring that not all ANCA positivity represents true AAV [7, 12].

As cocaine is frequently adulterated with levamisole, which can induce a vasculitis‐like syndrome which includes neutropenia, purpura, and arthralgia [13, 14], any patient with non‐organ threatening or ENT‐limited disease should have urine toxicology assessments. Although urine toxicology has a limited detection window as cocaine metabolites typically clear within 72 h, patients who are chronic users of cocaine may have persistent positive urine toxicology [15]. However, a negative urine test also does not rule out recent use [15].

Another method that can help differentiate between CIMDL/CLAAS and true AAV in intermittent, long‐term users of cocaine is hair testing. Hair testing offers a more reliable measure of chronic exposure, including to levamisole. In the case by van der Veer et al. [13], hair testing provided critical diagnostic clarity when urine toxicology results were negative. This may prove of value especially since radiologically, CIMDL often shows destructive lesions in the nasal septum and turbinates, indistinguishable from GPA on imaging alone. Furthermore, histopathologic, CIMDL biopsies reveal necrotizing inflammation without the granulomatous features typical of GPA, though variability necessitates clinical correlation.

Double positivity to ANCA may also indicate that drug‐induced Vasculitides may be at play [16]. In the recent study by Bettacchioli et al., [16] the authors concluded that approximatively 50% of double positive‐ANCA patients develop AAV, either as drug‐induced or true idiopathic forms, while the remaining 50%, characterized by pre‐existing dysimmune conditions, which demonstrated a remarkably lower risk of developing significant vasculitis. This can be another potential discriminator of CIMDL/CLAAS rather than true AAV.

It is worth noting that it may be necessary to use immunosuppressive therapy in cases where systemic features predominate (Berman et al. 2016 [13]). While immunosuppression is generally ineffective in CIMDL, selected patients with CLAAS may benefit if systemic features are a dominant clinical phenotype. However, the default use of these therapies without confirming etiology must be avoided [5].

In this cohort, several patients had eosinophilia alongside the PR3‐ANCA antibodies. This is an unusual pairing in GPA and it is rather seen in Eosinophilic granulomatosis with polyangiitis (EGPA). These discrepancies demand broader differential diagnoses. Differential diagnoses such as hypereosinophilic syndromes, Hyper‐IgE syndromes, fungal and parasitic infections need to be excluded. Also malignancies (such as Hodgkin Lymphoma and T‐Cell Lymphoma) can have raised eosinophils as a paraneoplastic phenomenon. Therefore a high index of suspicion is required to ensure that all these differential diagnoses are excluded. Other drug exposure can also be a cause. Cohen Tervaert and Damoiseaux (2012) have emphasized that clinicians must interpret ANCA pattern and antigen specificity together, especially when drug exposure is suspected.

The presence of eosinophilia can further complicate diagnosis. All patients in this series had eosinophilia, which may suggest eosinophilic granulomatosis with polyangiitis or drug‐induced hypersensitivity [17]. Eosinophilic infiltration may mimic granulomatous vasculitis histologically and be misinterpreted as AAV [2, 18]. However, levamisole is known to have immunostimulatory properties which can contribute to eosinophilia in either CLAAS or CIMDL [14, 19].

To improve diagnostic accuracy, clinicians should implement structured protocols that include routine drug screening during the initial evaluation of suspected AAV, especially in patients with predominant sinonasal disease. Delayed toxicology testing not only leads to misdiagnosis but also misses the chance to intervene on substance misuse [5, 15]. The complexity of these presentations supports the need for multidisciplinary care, involving rheumatologists, otolaryngologists, pathologists, and addiction specialists [8].

Further research should quantify ANCA prevalence in drug‐using populations and explore immune responses to cocaine and levamisole. Large prospective studies comparing idiopathic AAV with CLAAS may reveal novel biomarkers, similar to elastase ANCA, that improve diagnostic discrimination [20]; (Blanco et al., 2022). Ultimately, the cessation of cocaine use is critical for favorable outcomes in CIMDL. Rossi et al. (2021) demonstrated that patients who discontinued cocaine achieved disease stabilization and significant symptom relief with prosthetic rehabilitation, while continued use led to progressive tissue damage and poor treatment response.

### 3.1 Study Limitation

This case series has several limitations that should be acknowledged. Firstly, the retrospective nature of the study introduces potential biases, including reliance on medical records for clinical data and the possibility of incomplete or missing information. Additionally, the identification of cocaine use was contingent upon patient self‐reporting and urine toxicology testing, which has a limited detection window. Consequently, chronic cocaine use that ceased before hospital presentation may not have been detected, potentially leading to misclassification of cases as idiopathic ANCA‐associated vasculitis (AAV).

Secondly, while the study emphasizes the diagnostic overlap between cocaine/levamisole‐associated autoimmune syndrome (CLAAS) and AAV, the absence of elastase ANCA testing is a notable limitation. Elastase antibodies may serve as a more specific marker for drug‐induced vasculitis and could have provided further diagnostic clarity, particularly in patients with atypical ANCA patterns or persistent PR3 positivity despite cessation of drug use.

Furthermore, histopathologic analysis was not uniformly conducted across all cases, limiting the ability to draw comprehensive conclusions about the presence or absence of necrotizing granulomatous inflammation or eosinophilic infiltrates. The absence of standardized histological examination protocols may have contributed to variability in diagnostic interpretation.

Another limitation is the small sample size of seven patients, which restricts the generalizability of findings. Given the rarity of CIMDL and CLAAS, larger multicenter studies are needed to better delineate clinical, radiologic, and immunologic distinctions between drug‐induced and idiopathic AAV.

Finally, the study did not include hair testing for cocaine metabolites or levamisole, which could have provided a more extended detection window and potentially identified additional cases of chronic drug exposure missed by urine toxicology alone. This limitation underscores the need for standardized substance abuse screening protocols in the diagnostic workup of patients with suspected AAV or drug‐induced vasculitis.

## 4 Conclusion

This case series demonstrates the diagnostic complexity surrounding CLAAS and CIMDL, particularly in patients with ANCA positivity and sinonasal symptoms. Misdiagnosis as idiopathic ANCA‐associated vasculitis (AAV) can lead to unnecessary and potentially harmful immunosuppressive treatment.

Accurate diagnosis depends on careful ANCA interpretation, awareness of atypical serologic patterns, and systematic toxicology screening. Clinicians must recognize the immunological effects of cocaine and levamisole, especially amid rising recreational drug use. A structured, multidisciplinary approach—including addiction assessment—can prevent misdirected treatment and ensure patients receive care aligned with their underlying pathology.

## Author Contributions

**Kehinde Sunmboye:** conceptualization, investigation, writing – original draft, methodology, writing – review and editing, data curation, supervision, resources. **Ameen Jubber:** data curation, project administration, resources, visualization, writing – review and editing. **Maumer Durrani:** data curation, resources, project administration, visualization, writing – review and editing. **Jeremy Royle:** data curation, resources, project administration, visualization, methodology. **Shireen Shaffu:** data curation, resources, project administration, formal analysis, writing – review and editing, visualization.

## Ethics Statement

Ethics committee approval was received for this study under the Leicester, Leicesteshire and Rutland ethics committee of Institutional Review Board Number 12470a.

## Consent

Consent was obtained from patients who participated in this study.

## Conflicts of Interest

The authors declare no conflicts of interest.

## Data Availability

The authors have nothing to report.
